# Jaccard index based similarity measure to compare transcription factor binding site models

**DOI:** 10.1186/1748-7188-8-23

**Published:** 2013-09-30

**Authors:** Ilya E Vorontsov, Ivan V Kulakovskiy, Vsevolod J Makeev

**Affiliations:** 1 Laboratory of Bioinformatics and Systems Biology, Engelhardt Institute of Molecular Biology, Russian Academy of Sciences, Vavilov str. 32, Moscow 119991, GSP-1, Russia; 2 Department of Computational Systems Biology, Vavilov Institute of General Genetics, Russian Academy of Sciences, Gubkina str. 3, Moscow 119991, GSP-1, Russia; 3 Data Analysis Department, Yandex Data Analysis School, Moscow Institute of Physics and Technology, Leo Tolstoy str. 16, Moscow 119021, Russia; 4 Department of Biological and Medical Physics, Moscow Institute of Physics and Technology, Institutskiy per. 9, Dolgoprudny 141700, Moscow Region, Russia

**Keywords:** Transcription factor binding site, TFBS, Transcription factor binding site model, Binding motif, Jaccard similarity, Position weight matrix, PWM, P-value, Position specific frequency matrix, PSFM, Macroape

## Abstract

**Background:**

Positional weight matrix (PWM) remains the most popular for quantification of transcription factor (TF) binding. PWM supplied with a score threshold defines a set of putative transcription factor binding sites (TFBS), thus providing a TFBS model.

TF binding DNA fragments obtained by different experimental methods usually give similar but not identical PWMs. This is also common for different TFs from the same structural family. Thus it is often necessary to measure the similarity between PWMs. The popular tools compare PWMs directly using matrix elements. Yet, for log-odds PWMs, negative elements do not contribute to the scores of highly scoring TFBS and thus may be different without affecting the sets of the best recognized binding sites. Moreover, the two TFBS sets recognized by a given pair of PWMs can be more or less different depending on the score thresholds.

**Results:**

We propose a practical approach for comparing two TFBS models, each consisting of a PWM and the respective scoring threshold. The proposed measure is a variant of the Jaccard index between two TFBS sets. The measure defines a metric space for TFBS models of all finite lengths. The algorithm can compare TFBS models constructed using substantially different approaches, like PWMs with raw positional counts and log-odds. We present the efficient software implementation: MACRO-APE (MAtrix CompaRisOn by Approximate P-value Estimation).

**Conclusions:**

MACRO-APE can be effectively used to compute the Jaccard index based similarity for two TFBS models. A two-pass scanning algorithm is presented to scan a given collection of PWMs for PWMs similar to a given query.

**Availability and implementation:**

MACRO-APE is implemented in ruby 1.9; software including source code and a manual is freely available at http://autosome.ru/macroape/ and in supplementary materials.

## Background

Transcription factors (TFs) with similar structures of their DNA binding domains often recognize similar transcription factor binding sites (TFBS). TF binding DNA segments obtained by different experimental techniques can be systematically different even for the same TF. Different motif discovery algorithms applied to the same set of TF binding sequences usually produce different results [1]. Thus, the problem of comparing transcription factor binding models arises in different contexts. The typical representation of a TF-recognized DNA binding pattern is a positional weight matrix (PWM, or position specific frequency matrix, PSFM). When PWM is used to predict TFBS in DNA sequence, different score cutoffs (thresholds) result in different sets of tentative TFBS. The complete set of tentative TFBS is defined by a TFBS model as a combination of a PWM and its score threshold.

A number of methods have been developed to measure similarity of two PWMs. The basic approaches were proposed more than 10 years ago [2,3]. A number of practical implementations were developed [4-11], with many of them included in integrated tools [12]. Most of these methods rely on comparison of PWM elements computing, e.g., the correlation between matrix elements at particular TFBS positions. From a practical standpoint, it seems more relevant to compare the sets of tentative TFBS recognized by PWMs at given threshold levels rather than the PWMs *per se*. Indeed, PWM thresholds selected in practice are usually high and, thus, the scores of tentative TFBS are close to the maximal PWM scores; only the matrix elements with high values contribute to the score of a putative TFBS. The matrix elements with low values rarely or almost never contribute to tentative TFBS scores, but contribute to the matrix similarity measures on par with PWM elements having high values, e.g., in case of the Pearson correlation computed for columns of two compared PWMs. For comparing the matrices with strictly positive values, e.g., counts of frequencies, this effect may be less important, but a log-odds PWM can contain negative elements with rather high absolute values, which would substantially bias the comparison.

Moreover, when the threshold values are high, two PWMs can predict the same set of tentative TFBS; but when score threshold levels are lower, the predicted TFBS sets may be rather different. Thus, it would be useful to have a similarity measure based not only on PWMs but also on threshold values.

The similarity measure for two PWMs, taking into account their thresholds, was first introduced in MoSta [13], which computes the correlation between the numbers of hits of two PWMs in a random DNA sequence. MoSta uses non-normalized matrices of integer letter counts. Still, in practice the PWMs are used along with different normalization strategies [14], e.g., commonly used log-odds transformation of counts [15], with resultant matrix elements having any real value. In addition, it seems more intuitive to have a similarity measure directly based on the number of binding sites recognized by both tested TFBS models.

Here we propose a measure based on the Jaccard similarity index to evaluate the similarity of two sets of possible TFBS defined by two PWMs with respective threshold values. For two PWMs taken with their thresholds, this measure can be used to obtain the optimal PWM alignment, i.e., the displacement (shift) of the first PWM relative to the second, at which they recognize the most similar sets of TFBS. We show that the suggested measure defines a metric space on a set of binding models of TFBS of any finite length, considering TFBS generated by the Bernoulli (*i.i.d.*) random model.

The paper is organized as follows: the Algorithm section presents a basic introduction into the problem followed by the formal construction of the proposed similarity measure; the Results and Discussion section presents validation of the proposed approach using the pairs of TFBS models for the same TF; the Conclusions section contains the final remarks; proofs of lemmas and a theorem introduced in the paper are given in the Appendix.

## Algorithm

The combination of a PWM and its score threshold makes up a TFBS model; the model defines some finite set of TFBS. Let us consider two models, *X* and *Y*, defining two sets of binding sites, **X** and **Y**, of the same length (width) at given threshold levels. One can directly apply the Jaccard measure to estimate the similarity between these two models:

$$J\left(X,Y\right)=\frac{\left|X\cap Y\right|}{\left|X\cup Y\right|}$$

where |**X**| is the size of the set **X** of binding sites defined by the model *X*. *J* is the fraction of words recognized by both models (i.e. scoring as no less than the corresponding thresholds for both PWMs) in the larger set of words recognized by any of the two models. It has already been shown [16] that this measure defines a metric space on the sets of words of the same length based on the distance:

$$D\left(X,Y\right)=1-J\left(X,Y\right)$$

Technically, |**X**| and |**Y**| can be computed using the existing approach [17] and |**X** ∪ **Y**| = |**X**| + |**Y**| − |**X** ∩ **Y**|, so the trick is to estimate |**X** ∩ **Y**|.

In general binding site lengths and strand orientations at the DNA heteroduplex may be different. Two TFBS models can be aligned by PWM shifting and possible reverse complement transformation. It is intuitively consistent that, if a longer model is compared with a shorter model, any symbol may occupy the “hanging positions” of the longer model. For the large shifts, both models can have “hanging positions” at the opposite ends. The similarity between the two models is defined as the maximal similarity attained after testing all possible relative shifts and orientations of the two respective PWMs. Below we prove that this measure maintains its metric properties for the TFBS models made up from PWMs and score thresholds. Moreover, we prove that the suggested similarity measure is applicable in a more general case of weighted contribution of different binding sites, e.g., with probabilistic weights based on an *i.i.d.* random model.

### General remarks

Our algorithm was inspired by the ideas of Touzet and Varre [17]. Let there be a sequence written in the alphabet *A* = {A, C, G, T}. Let us consider a PWM, a 4-by-*m* matrix *M:M* = [*M*(*α*, *i*)]_4 *m*_ with DNA positions at columns and DNA alphabet symbols at rows; *m* is the PWM width (the binding site length). $M\left(\alpha ,i\right)\in R$ represents a score at *i-*th position, 1 ≤ *i* ≤ *m*, for the letter *α* ∈ *A*. For each word *ω* = *ω*_1_.. *ω*_*m*_ in *A*^*m*^, this matrix defines a score:

$$S\left(\omega ,M\right)=\overset{m}{\underset{i=1}{\sum }}M\left(\omega_{i},i\right)$$

Given a threshold *t*, the PWM defines a motif occurrence in the sequence ζ at position *n* if *S*(*ζ*_*n*_.. *ζ*_*n* + *m* − 1_, *M*) ≥ *t*. A pair of a PWM and a threshold defines the TFBS recognition model allowing one to explicitly enumerate the set of all *m*-mers identified as TFBS:

$$\Omega \left(M,t\right)=\left\{\omega \in A^{m}:S\left(\omega ,M\right)\geq t\right\}$$

The P-value(*M, t*) is the probability *P*(*M*, *t*) that a background random model would generate a word with the score of no less than the threshold *t*:

$$P‐\text{value}\left(M,t\right)=P\left(M,t\right)=\underset{\omega \in \Omega \left(M,t\right)}{\sum }P\left(\omega \right),$$

where *P*(*ω*) is the probability of the word *ω* under the given background model.

Following [17], we define the *score distribution Q*(*M*, *s*) as the probability that the background model would generate a word *ω* with the exact score *s*. Formally,

$$Q\left(M,s\right)=\underset{\omega :S\left(\omega ,M\right)=s}{\sum }P\left(\omega \right).$$

If *s* is not an accessible score for the given PWM *M*, then *Q*(*M*, *s*) = 0. Knowing the score distribution, one can easily calculate the P-values:

$$P\left(M,t\right)=\underset{s\geq t}{\sum }Q\left(M,s\right)$$

### Zero-columns extension of PWM

**Lemma 1.** Extending a PWM with any number of zero columns from the left or from the right does not change the score distribution or any P-value corresponding to any score threshold.

### Reverse complement transformation of PWM

*Reverse complement transformation* of PWM *M* is a new PWM $\tilde{M},$ for which the following relations are valid for any column *i*:

$$\begin{array}{l} \tilde{M}\left(A,i\right)=M\left(T,m-i+1\right);\tilde{M}\left(T,i\right)=M\left(A,m-i+1\right);\\ \tilde{M}\left(C,i\right)=M\left(G,m-i+1\right);\tilde{M}\left(G,i\right)=M\left(C,m-i+1\right). \end{array}$$

Reverse complement transformation of a PWM is a PWM that locates the same set of TFBS but on the opposite strand of a DNA heteroduplex.

**Lemma 2.** If the words are generated by an *i.i.d.* random model and the background probabilities comply with the conditions *p*(A) = *p*(T), *p*(C) = *p*(G), then the reverse complement transformation of PWM *M* does not change the score distribution and hence the P-values.

### Alignment of PWMs of different widths

Suppose there are two PWMs, *M*_1_ and *M*_2_, of possibly different widths *m*_1_,*m*_2_, applied to some sequence ζ starting from positions *j*_1_,*j*_*2*_, respectively. When written with any relative shift, these two matrices can be appended with zero columns at all non-aligned (“hanging”) positions. To be more precise, two matrices can be *aligned* by extending *M*_1_ with zero columns at all positions overlapping with *M*_2_ but not with *M*_1_, and by extending *M*_2_ with zero columns at all positions overlapping with *M*_1_ but not with *M*_2_. The aligned matrices have the same width *m* and define scores for the same dictionary of words.

The respective P-values can be calculated for the two aligned PWMs *M*_1_,*M*_2_ with thresholds *t*_1_,*t*_2_:

$$\begin{array}{l} P‐\text{value}\left(M_{1},t_{1}\right)=\underset{s\geq t_{1}}{\sum }Q\left(M_{1},s\right)=P\left(\Omega_{1}\left(M_{1},t_{1}\right)\right);\\ P‐\text{value}\left(M_{2},t_{2}\right)=\underset{s\geq t_{2}}{\sum }Q\left(M_{2},s\right)=P\left(\Omega_{2}\left(M_{2},t_{2}\right)\right), \end{array}$$

where Ω_1_,Ω_2_ are the word sets defined by the corresponding PWMs *M*_1_,*M*_2_ with thresholds *t*_1_,*t*_2_.

The similarity measure of word sets Ω_1_,Ω_2_ and thus of the models defined by *M*_1_ and *M*_2_ used with the thresholds *t*_1_,*t*_2_ is computed as the conditional probability that a random word *ω* has scores no less than the preselected thresholds for both matrices, knowing that its score is no less than the corresponding threshold for at least one of the two matrices:

$$J1\left(\Omega_{1},\Omega_{2}\right)=\frac{P\left(\left\{\omega :\omega \in \Omega_{1}\cap \Omega_{2}\right\}\right)}{P\left(\left\{\omega :\omega \in \Omega_{1}\cup \Omega_{2}\right\}\right)}.$$

In case of uniform probability distribution, *p*(α) = 0.25 for all *α* ∈ *A*, this measure is simplified to the ratio of the number of words scoring no less than the thresholds for both matrices and the number of words scoring no less than the corresponding threshold for any of the matrices:

$$J1\left(\Omega_{1},\Omega_{2}\right)=\frac{\left|\Omega_{1}\cap \Omega_{2}\right|}{\left|\Omega_{1}\cup \Omega_{2}\right|},$$

which coincides with the Jaccard similarity measure for two sets of words.

The distance *D*1(Ω_1_, Ω_2_) = 1 − *J*1(Ω_1_, Ω_2_) is a metric on the weighted word sets [16]. In our example, the weights of words are derived as their probabilities to be generated by an *i.i.d.* random model.

**Lemma 3.** Let there be an aligned pair of PWMs *M*_1_,*M*_2_ with the corresponding thresholds *t*_1_,*t*_2_, defining TFBS recognition models Ω_1_,Ω_2_. Extension of both PWMs with any number of zero columns does not change *D*1(Ω_1_, Ω_2_).

### Definition of the distance metric for TFBS models

Let us finally define the distance between the two unaligned recognition models Ω_1_,Ω_2_ represented as PWMs *M*_1_,*M*_2_ of possibly different widths *m*_1_,*m*_*2*_ with the given thresholds *t*_1_,*t*_2_ corresponding to P-values *P*_1_ = *P*(*M*_1_, *t*_1_), *P*_2_ = *P*(*M*_2_, *t*_2_):

$$\Omega_{1}=\Omega \left(M_{1},t_{1}\right)\;\mathrm{and}\;\Omega_{2}=\Omega \left(M_{2},t_{2}\right).$$

Close PWMs at close P-values identify similar sets of DNA words on any of the two strands of DNA heteroduplex. Two PWMs can be aligned with any relative shift. In addition, one of PWMs can undergo reverse complement transformation. In so doing, the similarity between two PWMs can be defined as the maximal similarity attained after testing all possible shifts and orientations:

$$J2\left(\Omega_{1},\Omega_{2}\right)=\underset{i}{\text{max}}\left(\text{max}\left(J1_{i}\left(\Omega_{1},\Omega_{2}\right),J1_{i}\left(\Omega_{1},{\tilde{\Omega }}_{2}\right)\right)\right),$$

and similarly, the distance is defined as

$$D2\left(\Omega_{1},\Omega_{2}\right)=\underset{i}{\text{min}}\left(\text{min}\left(D1_{i}\left(\Omega_{1},\Omega_{2}\right),D1_{i}\left(\Omega_{1},{\tilde{\Omega }}_{2}\right)\right)\right).$$

Here, *J*1_*i*_(Ω_1_, Ω_2_) is the similarity between TFBS binding models based on PWMs *M*_1_,*M*_2_ aligned in such a way that the 1-st column of the matrix *M*_1_ corresponds to the (1+i)-th column of the matrix *M*_2_, 1 − *m*_1_ ≤ *i* ≤ *m*_2_ − 1, with the positive values of *i* corresponding to *M*_1_ extended from the left (and *M*_*2*_ extended from the right) and ${\tilde{\Omega }}_{2}$ being the TFBS model constructed with the reverse complement transformation of *M*_2_. Note that *J*2 defines the optimal alignment and the mutual orientation of the PWMs *M*_1_,*M*_2_ at the given thresholds *t*_1_,*t*_2_.

**Theorem:** Distance *D*2(Ω_1_, Ω_2_) = 1 − *J*2(Ω_1_, Ω_2_) defines a proper metric in the space of TFBS models represented as PWMs with thresholds corresponding to the given P-value levels.

Please see the Appendix for the proof.

### Calculating the size and the probability of a word set recognized by two models

Let us have two PWMs of the same width *m* with selected thresholds defining word sets Ω_1_ and Ω_2_. To compute *J*2, we need to estimate |Ω_1_ ∩ Ω_2_|, |Ω_1_ ∪ Ω_2_|, where |Ω_1_ ∪ Ω_2_| = |Ω_1_| + |Ω_2_| − |Ω_1_ ∩ Ω_2_| (a similar expression holds for weighted words, e.g., using the probabilities to be generated by an *i.i.d.* random model). The size of each of the word sets Ω_1_ and Ω_2_ recognized by the first and the second matrix at the given thresholds, or the probabilities *P*({*ω* : *ω* ∈ Ω_1_}), *P*({*ω* : *ω* ∈ Ω_2_}) in case of weighted words, can be calculated using the strategy described in [17]. So the remaining task is to calculate |Ω_1_ ∩ Ω_2_| or *P*({*ω* : *ω* ∈ Ω_1_ ∩ Ω_2_}).

The size of the word set Ω_1_ ∩ Ω_2_ can be calculated using a dynamic programming approach in a way similar to that in [13]. Let *S*_1_ and *S*_2_ be the PWM scores of some word prefix of length *i* ≤ *m* for PWMs *M*_1_ and *M*_2_, respectively. We maintain a two-dimensional hash *H*(*S*_1_, *S*_2_), where each key is the pair of scores (*S*_1_,*S*_2_) and each value is the number of prefixes of a given length having this pair of scores.

Having the hash *H*_*i*_ for the prefix length *i*, we can recalculate the hash for the (*i*+1)-th step:

Hi+1S1',S2'=∑α∈A,C,G,T∑S1:S1+M1α,i+1=S1'∑S2:S2+M2α,i+1=S2'HiS1,S2.

Having *H*_*m*_ for the full PWM width *m,* we can now calculate the size of the set Ω_1_ ∩ Ω_2_:

$$\left|\Omega_{1}\cap \Omega_{2}\right|=\underset{S_{1}\geq t_{1};S_{2}\geq t_{2}}{\sum }H_{m}\left(S_{1},S_{2}\right).$$

In case of words generated by an *i.i.d.* random model, the following formula can be used to calculate *H*_*i*+1_ which, in turn, will be storing the probabilities of generating prefixes with a given pair of scores:

Hi+1S1',S2'=∑α∈A,C,G,T∑S1:S1+M1α,i+1=S1'∑S2:S2+M2α,i+1=S2'HiS1,S2⋅pα

where *p*_*α*_, *α* ∈ {A, C, G, T} are the background probabilities of individual letters.

## Results and discussion

PWM based TFBS models are extensively applied in regulatory genomics. The existing TFBS models are stored in many different model collections and databases, e.g., proprietary TRANSFAC [18], or open access JASPAR [19], or recently published integrative HOCOMOCO [20]. These collections contain hundreds of PWMs for TFs of different structural families. PWMs for the same TF stored in different databases are usually obtained from different experimental data and/or using different motif discovery tools. The question of practical interest is to estimate a degree of similarity between the sets of binding sites defined by different models for the same TF.

To this end, we have selected 85 pairs of PWMs for TFs with the models present both in JASPAR and HOCOMOCO. We applied MACRO-APE to estimate the similarities between the models for a set of P-values each time specifying the same P-value for both compared PWMs. It would be logical to specify the same P-value for both PWMs, because it ensures that the sets of words independently recognized by each matrix are comparable in size.

**Figure 1:**
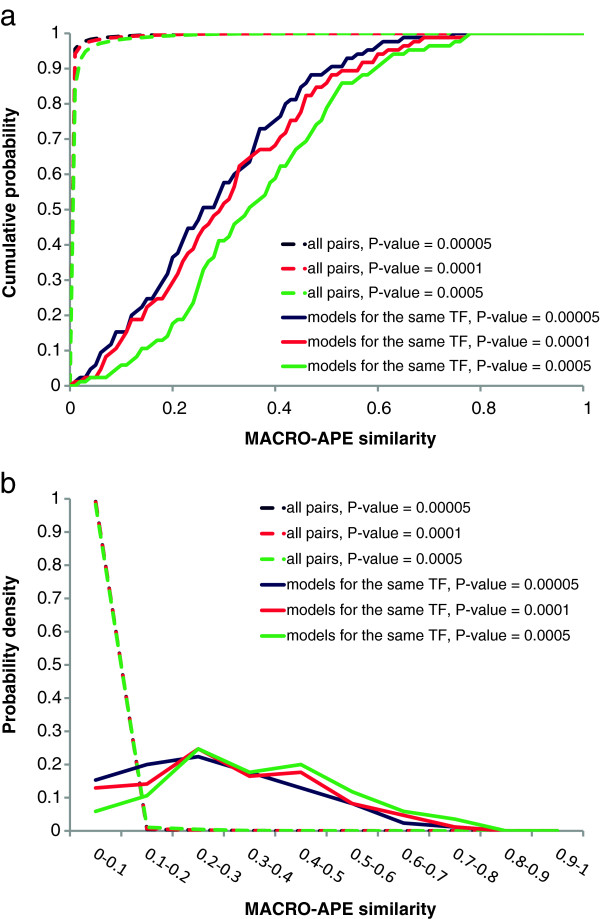
**The cumulative distributions (*a*) and probability density (*b*) of similarities for pairs of TFBS models.** The similarities for pairs of models for the same TF are shown by solid lines (data for 85 TFs with the models available in both HOCOMOCO [20] and JASPAR [19] databases). The similarities for all possible pairs for 170 assessed models are shown by dashed lines. Different colors correspond to different P-value levels. It is notable that the paired models for the same TF are really closer as compared with the whole set of possible pairs.

Figure 1 shows the distributions of similarity for the pairs of TFBS models for the same TF and for all possible pairs of models. The models for the same TF are indeed much more similar than all other non-matched pairs of models. Moreover, in general the average similarity of models for the same TF only weakly depends on the P-value (PWM threshold) selected for testing. The above confirms the relevance of our metric and indicates that in practice it is mostly safe to vary the P-value (and thus the positive TFBS prediction rate of the model) in a wide range of values. On the other hand, the absolute similarity level for a pair of models for the same TF indicates a rather low number (30-50%) of binding sites being shared. Thus, two sets of TFBS predicted in DNA sequence by different models obtained in different public sources can be really different from each other, which additionally confirms that appropriate choice of the model can be of profound importance for real-life genomic studies.

**Figure 2:**
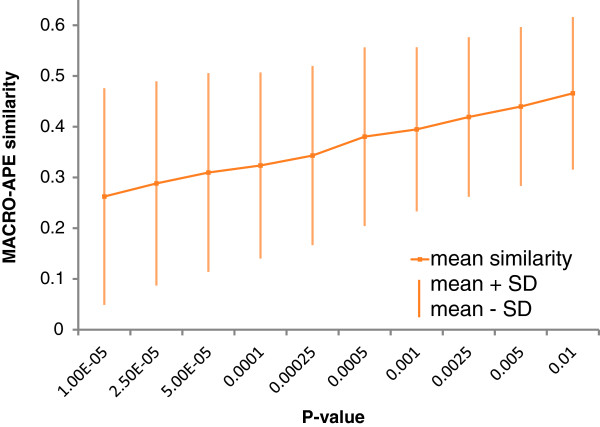
**The mean and the standard deviation of similarities between TFBS models for the same TF.** Similarities are computed for HOCOMOCO and JASPAR TFBS models for 85 TFs.

**Figure 3:**
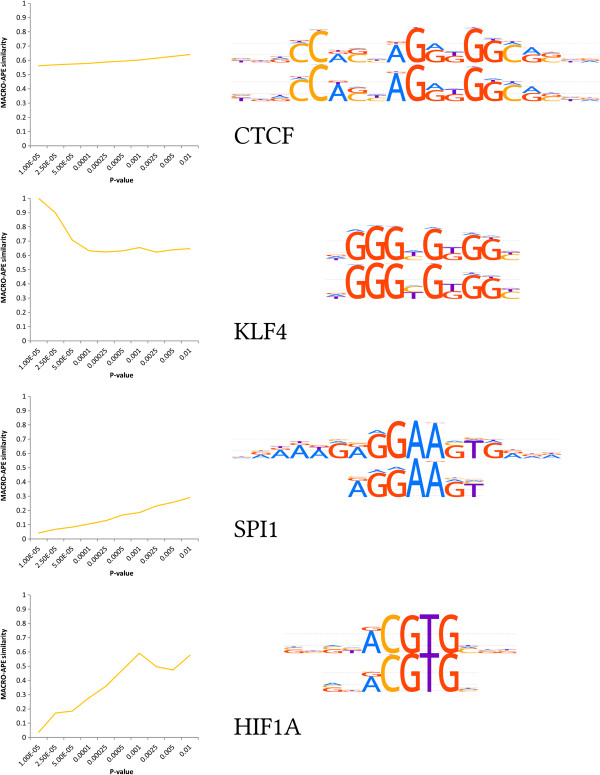
**The similarities (depending on P-value) and LOGO representations for pairs of TFBS models (HOCOMOCO and JASPAR) for selected TFs.** It is notable that even for extremely similar LOGOs, like those of CTCF, the Jaccard similarity reaches only 0.6, indicating that the models define the sets of binding sites overlapping only for 60%. The similarity remains comparatively low even at high P-values (e.g. 0.01 where each 100^th^ word of the dictionary is recognized as the binding site). The same effect is shown for KLF4 (with the exception of similarity 1.0 for the lowest P-value, where both models recognize only identical consensus sequences). SPI1 models differing in length show very weak similarities. HIF1A models are surprisingly dissimilar at low P-values (possibly due to shorter model lengths).

Figure 2 shows the mean and the standard deviation of similarities calculated for the pairs of models of the same TF depending on the P-value used for both PWMs. It is notable that the variance of similarity in the region of medium and high P-values is very stable. In practice, lower P-values are often selected to minimize false positive predictions. In this region, the similarity values vary greatly from one TF to another, which is accompanied with the decreased mean similarity, thus indicating even less stable TFBS predictions between different models for the same TF. Figure 3 shows the results for several selected pairs of the models with their motif LOGO representations. It is notable that even CTCF TFBS models with almost identical LOGOs and very well defined TFBS have the Jaccard similarity of only 0.6. It corresponds to 60% of shared sites among those predicted by any of the two models, or about 80% of predictions of each single model.

To further illustrate specific features of the Jaccard similarity we have plotted a series of heatmaps displaying the Jaccard similarity versus the similarity defined by the averaged column-wise Pearson correlation of two PWMs (for the optimal PWM alignment). The heatmaps for different P-value levels are given in the Additional file 1. For a generic pair of PWMs the Jaccard similarity is typically close to zero, while the Pearson correlation is positive and can be up to 0.3 – 0.5. For pairs of PWMs for the same TF the Jaccard similarity mostly has positive values. Yet there are many cases showing high Pearson correlation and low Jaccard similarity, meaning that highly correlated matrices may actually correspond to TFBS models recognizing quite different word sets (as we hypothesized in the Background section).

**Figure 4:**
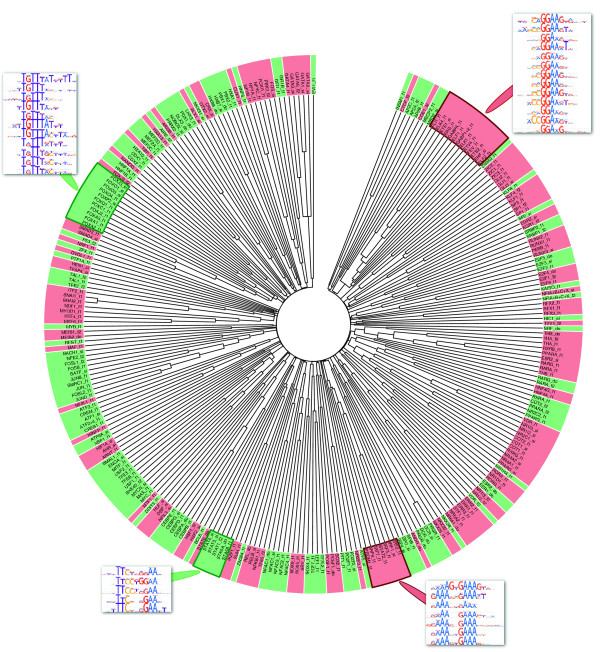
**The circular tree illustrating the hierarchy of high quality models from HOCOMOCO collection.** Clusters are shown by alternating colors. The examples of clustered TFBS models are shown with respective LOGO representations. The tree is drawn using jsPhyloSVG [22].

We have also applied MACRO-APE to classify TFBS models of different TFs. Using the Jaccard similarity we produced an UPGMA linkage tree [21] for high quality PWMs of the HOCOMOCO TFBS model collection [20]. The P-value level of 0.0005 was adopted for all PWMs. The corresponding pairwise similarity matrix is provided in the Additional file 2. The clusters were naturally obtained by gathering PWMs on the same branch while traversing the tree. The algorithm was terminated when the maximal value of pairwise distance between the cluster elements became higher than 0.95 (i.e., when the minimal pairwise similarity between cluster elements became lower than 0.05, in other words, when two most dissimilar PWMs in the cluster shared less than 5% of words among the words recognized by any of these PWMs). Figure 4 shows the circular tree illustrating the hierarchy of PWMs from the HOCOMOCO collection.

### Technical notes

The algorithm running time is proportional to the product of the numbers of possible different scores for *M*_1_ and *M*_2_, being $O\left(4^{m_{1}+m_{2}}\right)$ in the worst possible case. The algorithm complexity is dramatically decreased by PWM discretization strategy as in [17]. For the PWM element *v* we define discretized *v*’ as *v* multiplied by the discretization level *d* and rounded up to the nearest integer value. In contrast to the original Touzet’s approach, we apply “ceil” operation to each PWM element during discretization so that to obtain the upper boundary of the threshold for the given P-value.

Discretization generally maintains word ranking, but at lower discretization levels more words receive identical scores. The effective number of different scores is decreased to the value of

$$\begin{array}{l} \text{max}\_\text{discrete}\_\text{score}-\text{min}\_\text{discrete}\_\text{score}\\ \;=O\left(\left(\text{max}\_\text{score}-\text{min}\_\text{score}\right)\cdot d\cdot m\right). \end{array}$$

Thus, the overall complexity of the |Ω_1_ ∩ Ω_2_| calculation algorithm would be:

$$O\left({\left(\left(\text{max}\_\text{score}-\text{min}\_\text{score}\right)\cdot d\cdot m\right)}^{2}\right).$$

In case of PWMs of different widths and unknown mutual orientation, all possible alignments are to be checked; hence, the overall complexity is cubic relative to the PWM width like *O*(*m*^3^). The algorithm can be further improved by early discarding the hash elements that cannot exceed the given threshold even for the best available suffix [17].

We have implemented the algorithm for the popular PWM model using P-values estimated for an *i.i.d.* random model. The real genomic sequences almost never comply with an *i.i.d.* assumption. Nevertheless, PWMs stored in the existing databases are often constructed from the binding sites in genomic sequences of very different nucleotide composition (for instance, those extracted from genomes of different species). Some *in vitro* experimental methods, e.g., parallel SELEX [23] or protein-binding microarrays [24], provide a huge dictionary of purely synthetic random DNA oligonucleotides evaluated for their affinity as binding sites to a particular protein. So, the suggested variant of the Jaccard measure seems to be useful for practical application even taking into account the very basic TFBS and background models.

At the same time, the measure seems to be extensible for more complex models such as the 1^st^ order Markov chains. The background model also can be generalized to use Markov model assumption. Unfortunately algorithm complexity grows exponentially as *O*(4^*k*^) with Markov chain order *k* so that the construction of the appropriate software tool for large-scale analysis remains a challenge.

## Conclusions

The MACRO-APE software allows computing the Jaccard similarity measure for a pair of PWMs with given threshold values. The proposed approach reveals critical differences in the sets of binding sites defined by the commonly used TFBS models. The software allows scanning a given collection of matrices for PWMs similar to a given query at given score thresholds or P-value levels. We have implemented a two-pass scanning tool, which quickly filters out dissimilar entries and then carefully processes a smaller set of candidate models. Along with these tools, MACRO-APE provides basic utilities to estimate a PWM threshold for a given P-value and vice versa. Software source code and user manual are provided as the Additional files 3 and 4.

## Availability and requirements

**Project name:** MACRO-APE (MAtrix СompaRisOn by Approximate P-value Estimation)

**Project home page:**http://autosome.ru/macroape/

**Operating system(s):** Platform independent

**Programming language:** Ruby

**Other requirements:** Ruby 1.9.3 or higher

**License:** MIT License

## Appendix: proofs of lemmas and main theorem

### Zero-columns extension of PWM

**Lemma 1.** Extending a PWM with any number of zero columns from the left or from the right does not change the score distribution or any P-value corresponding to any score threshold.

**Proof:** It is enough to have a proof for a single column appended from the right. A new extended matrix [*M*_*E*_]_4 * (*m* + 1)_ defines the scores for *ω* ∈ *A*^*m* + 1^. For the zero column, *M*[*α*, *m* + 1] = 0 for all *α* in *A* and *S*(*ω*, *M*_*E*_) = *S*(*ω*[1.. *m*], *M*). P-value can be calculated from the score distribution: $P\left(M_{E},t\right)=\underset{s\geq t}{\sum }Q\left(M_{E},s\right)$.

The word set *Ω*_*E*_ = {*ω* ∈ *A*^*m* + 1^ : *S*(*ω*, *M*_*E*_) ≥ *s*} can be obtained from the word set Ω by adding all 1-suffixes {*ω*[*m* + 1]} = *A* to any word *ω*[1.. *m*] from Ω. If words are generated by an *i.i.d.* random model, their probabilities are the products of the letter probabilities *p*(*α*). So the probabilities of (*m*+1)-mers in Ω factorize and the resulting probability does not change:

$$\begin{array}{l} Q\left(M_{E},s\right)=\underset{\omega \in \Omega_{E}}{\sum }P\left(\omega \right)=\underset{\omega \in \Omega_{E}}{\sum }P\left(\omega \left[1..m\right]\right)p\left(\omega_{m+1}\right)=\\ =\underset{\omega \in \Omega }{\sum }P\left(\omega \left[1..m\right]\right)\underset{\xi \in A}{\sum }p\left(\xi \right)=\underset{\omega \in \Omega }{\sum }P\left(\omega \left[1..m\right]\right)=Q\left(M,s\right). \end{array}$$

### Reverse complement transformation of PWM

**Lemma 2**. If the words are generated by an *i.i.d.* random model and the background probabilities comply with the conditions *p*(A) = *p*(T), *p*(C) = *p*(G) then the reverse complement transformation of PWM M does not change the score distribution and hence the P-values.

The assertion of this lemma directly follows from the definition of the score distribution after all substitutions made. For any word *ω* having a score *s* with *M* there is a corresponding hit with $\tilde{M}$, which is obtained as *ω* read backwards with substitutions A ⇔ T, G ⇔ C.

### Alignment of PWMs of different widths

**Lemma 3**. Let there be an aligned pair of PWMs *M*_1_,*M*_2_ with the corresponding thresholds *t*_1_,*t*_2_, defining TFBS recognition models Ω_1_,Ω_2_. Extension of both PWMs with any number of zero columns does not change *D*1 (Ω_1_,Ω_2_).

Proof: Again, it is enough to have a proof for a single column added from the right. The idea of the proof is very similar to that for Lemma 1. For the uniform probability distribution, let us consider the fraction $J1\left(\Omega_{1E},\Omega_{2E}\right)=\frac{\left|\Omega_{1E}\cap \Omega_{2E}\right|}{\left|\Omega_{1E}\cup \Omega_{2E}\right|}$. Ω_1*E*_ = Ω(*M*_1*E*_, *t*_1_) is obtained by adding all 1-suffixes to any word from Ω_1_ = Ω(*M*_1_, *t*_1_); the same is true for Ω_2*E*_ = Ω(*M*_2*E*_, *t*_2_). Thus, if a word is in Ω(*M*_1_, *t*_1_) ∩ Ω(*M*_2_, *t*_2_) then its four possible extensions are in Ω(*M*_1*E*_, *t*_1_) ∩ Ω(*M*_2*E*_, *t*_2_) and |Ω_1*E*_ ∩ Ω_2*E*_| = 4|Ω_1_ ∩ Ω_2_|.

All four 1-suffixes become added when transiting from (Ω_1_,Ω_2_) to (Ω_1*E*_,Ω_2*E*_). Thus any (*m*+1)-mer from Ω_1*E*_ or Ω_2*E*_ has a single corresponding *m-*mer in Ω_1_ ∪ Ω_2_ and for each *m-*mer in Ω_1_ ∪ Ω_2_ there are four (m+1)-mers in Ω_1*E*_ ∪ Ω_2*E*_. Thus |Ω_1*E*_ ∪ Ω_2*E*_| = 4|Ω_1_ ∪ Ω_2_|.

Reducing the fraction by 4 proves the lemma. In case of non-uniform background distribution of probabilities *p*_α_, it is important that the probability of an extended random word falling into Ω_1*E*_ ∩ Ω_2*E*_ is the same as for non-extended random word falling into Ω_1_ ∩ Ω_2_. The proof of the above is very similar to that of Lemma 1. The similar equation is true for the denominator, which proves the lemma.

### Definition of the distance metric for TFBS models

**Theorem:** Distance *D*2(Ω_1_, Ω_2_) = 1 − *J*2(Ω_1_, Ω_2_) defines a proper metric in the space of TFBS models represented as PWMs with thresholds corresponding to the given P-value levels.

**Proof**: To prove the theorem, one needs to demonstrate that *D*2 complies with the following metric properties:

1. *D*2(Ω_1_, Ω_2_) = 0 if and only if Ω_1_ = Ω_2_

2. *D*2(Ω_1_, Ω_2_) = *D*2(Ω_2_, Ω_1_)

3. *D*2(Ω_1_, Ω_2_) ≤ *D*2(Ω_1_, Ω_3_) + *D*2(Ω_2_, Ω_3_)

The second property is clear from the *D*2 definition and the first property follows from the observation that X ∩ Y = X ∪ Y only in the case when X=Y and the probability of a word set increases with the number of words. It only remains to prove the triangle inequality.

**Proof of the triangle inequality**. Note that the matrices become extended with zero-columns if necessary while the optimal shift and orientation are selected. This can be safely done according to Lemma 3. Thus, we omit the *E* index for matrices and models for simplicity.

Let us use the Ω_1|3_ notation for the model defined by *M*_1_ optimally aligned versus *M*_*3*_. We start from separate alignments of *M*_1_ and *M*_*2*_ with *M*_*3*_ as a reference. Thus we obtain two optimal alignments *M*_1_*vs M*_3_ and *M*_2_*vs M*_3_; the inherited alignment of *M*_1_*vs M*_2_ is not necessary optimal but conditioned by the respective optimal alignments with *M*_*3*_.

Nevertheless, all three matrices *M*_1_,*M*_*2*_,*M*_*3*_ become aligned, and for this alignment the triangle inequality is valid [16]:

$$D1\left(\Omega_{1|3},\Omega_{2|3}\right)\leq D1\left(\Omega_{1|3},\Omega_{3}\right)+D1\left(\Omega_{2|3},\Omega_{3}\right)$$

By construction, *D*1(Ω_1|3_, Ω_3_) = *D*2(Ω_1_, Ω_3_), and it is possible to rewrite the latter equation as *D*1(Ω_1|3_, Ω_2|3_) ≤ *D*2(Ω_1_, Ω_3_) + *D*2(Ω_2_, Ω_3_). Finally, by definition:

$$\begin{array}{l} D2\left(\Omega_{1},\Omega_{2}\right)=\min_{i}\left(\min\left(D1_{i}\left(\Omega_{1},\Omega_{2}\right),D1_{i}\left(\Omega_{1},{\tilde{\Omega }}_{2}\right)\right)\right)\\ \;\leq D1\left(\Omega_{1|3},\Omega_{2|3}\right) \end{array}$$

and, hence, *D*2(Ω_1_, Ω_2_) ≤ *D*2(Ω_1_, Ω_3_) + *D*2(Ω_2_, Ω_3_).

## Abbreviations

PWM: Position weight matrix; TF: Transcription factor; TFBS: Transcription factor binding site(s); UPGMA: Unweighted pair group method with arithmetic mean.

## Competing interests

The authors declare that they have no competing interests.

## Authors’ contributions

IEV implemented the software, participated in the algorithm development and manuscript preparation. IVK participated in the algorithm and software development, carried out testing and drafted the manuscript. VJM developed the initial algorithm, coordinated the software development process and helped finalize the manuscript. All authors have read and approved the final manuscript.

## Supplementary Material

Additional file 1 Density plots (heatmaps) of Pearson vs Jaccard similarity for generic PWM pairs and pairs of PWMs for the same TF. Click here for file

Additional file 2 Pairwise similarity matrix for high quality TFBS models of the HOCOMOCO collection. Click here for file

Additional file 3 **MACRO-APE source code (ruby 1.9).** Click here for file

Additional file 4 MACRO-APE user manual. Click here for file
