# Supplementary material for: Jaccard index based similarity measure to compare transcription factor binding site models
Source: Algorithms Mol Biol. 2013 Sep 30;8:23. doi: 10.1186/1748-7188-8-23 (PMC3851813; doi:10.1186/1748-7188-8-23)

**Additional file 1.** Density plots (heatmaps) of Pearson vs Jaccard similarity for generic PWM pairs and PWMs for the same TF. Color shows the fraction of pairs having given Jaccard (X axis) and Pearson (Y axis) similarities.


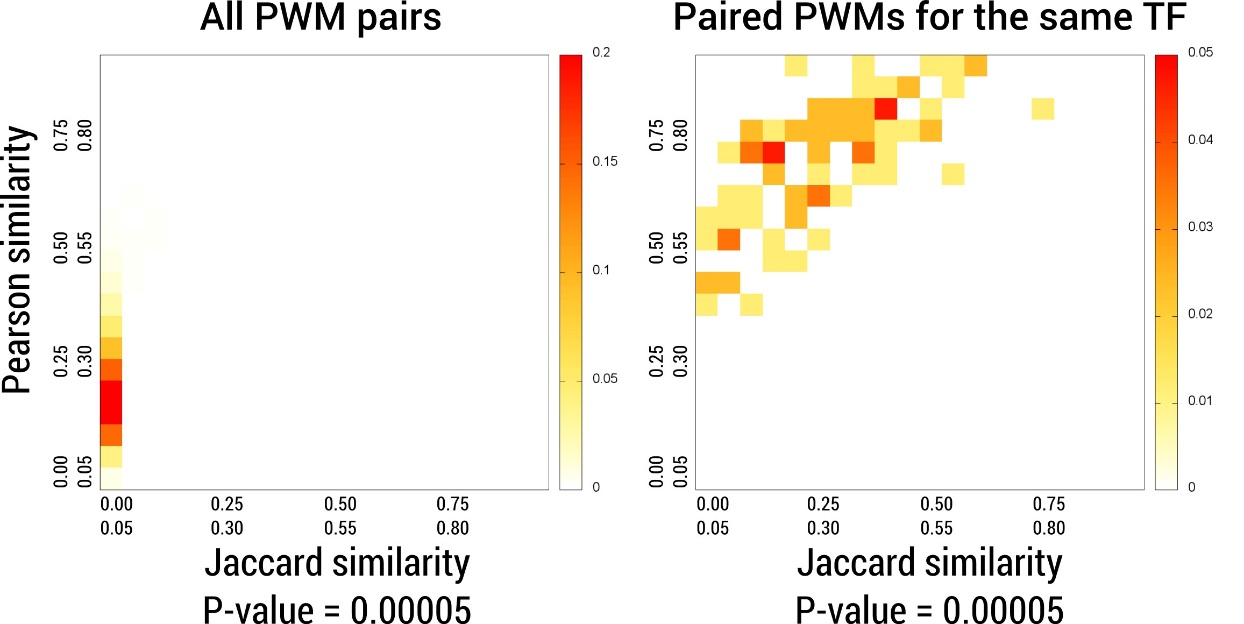


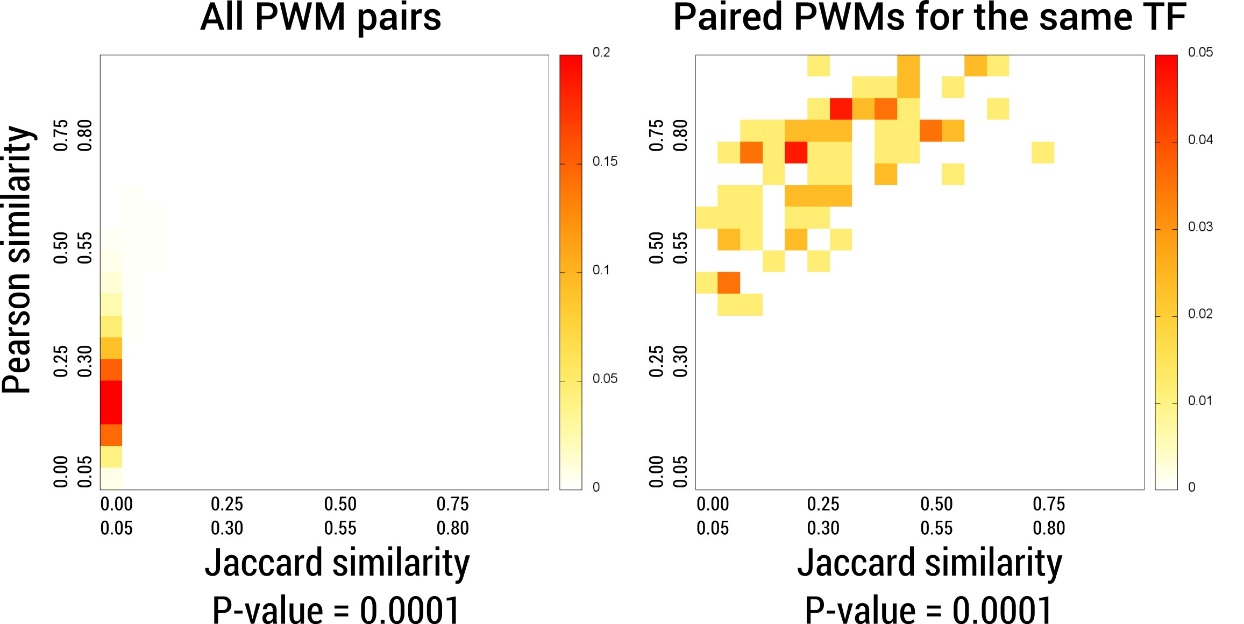


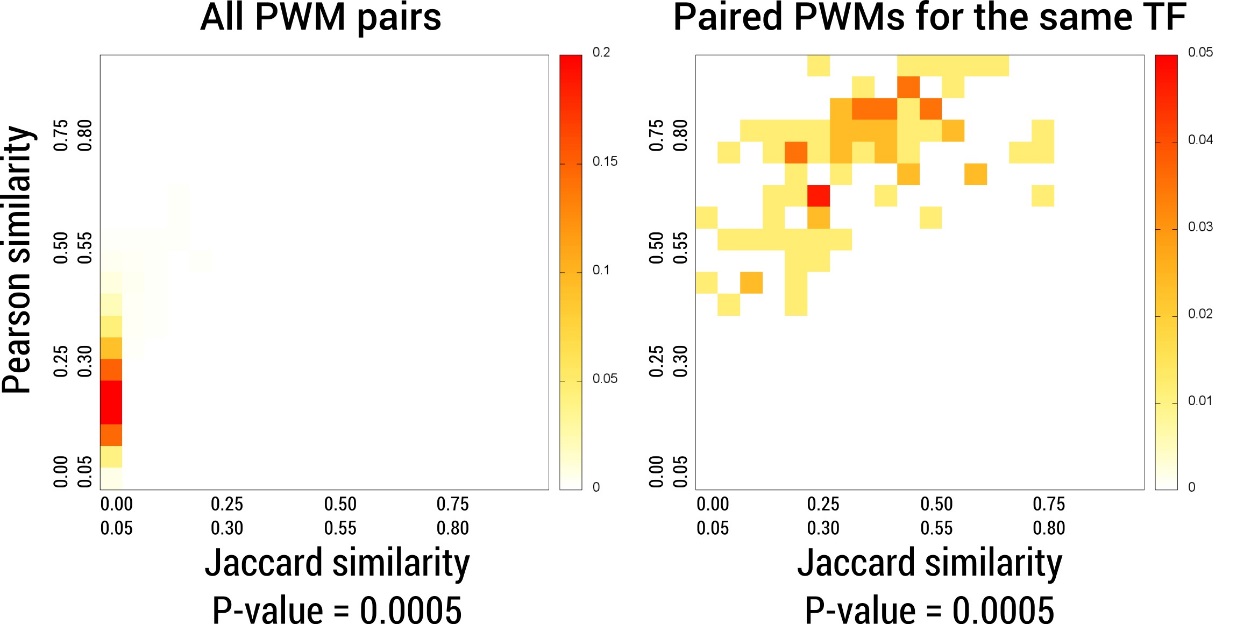

Supplement: Additional file 1 — Density plots (heatmaps) of Pearson vs Jaccard similarity for generic PWM pairs and pairs of PWMs for the same TF. [file 1748-7188-8-23-S1.docx]
