# Supplementary material for: Jaccard index based similarity measure to compare transcription factor binding site models
Source: Algorithms Mol Biol. 2013 Sep 30;8:23. doi: 10.1186/1748-7188-8-23 (PMC3851813; doi:10.1186/1748-7188-8-23)
Supplement: Additional file 4 — MACRO-APE user manual. [file 1748-7188-8-23-S4.pdf]

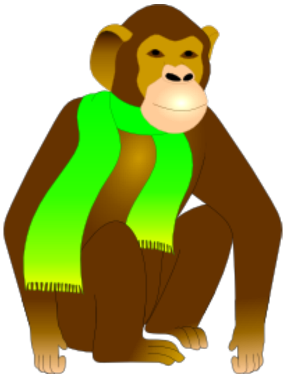

MACRO-APE

MAtrix **C**ompa**R**is**O**n by **A**pproximate **P**-value **E**stimation

- User Manual -

Abstract

Here we present the *macroape* software designed for effective comparison of transcription factor binding models (often called *motifs*) represented as position weight matrices (PWMs) with defined score thresholds.

Let us have two PWMs with given threshold levels. Using *macroape* software one can calculate the number of words recognized by both PWMs (or the aggregated probability of the word set under the given *i.i.d.* model). To calculate this value we use generalized approach described in **[Touzet2007]** for two PWMs simultaneously in a way similar to that in **[Pape2008]** . The number of words recognized by both PWMs can be used to construct a variant of Jaccard similarity measure for motifs considered as sets of allowed words.

Technical notes

macroape requires ruby 1.9.3 (or newer) to run. *macroape* works under Windows and Linux environments. With *macroape* we include preprocessed HOCOMOCO-AD [http://autosome.ru/HOCOMOCO] TFBS model collection and several examples of PWMs (motifs).

Windows users can get the latest ruby directly: [http://rubyinstaller.org/downloads/]. Linux users are advised to check the corresponding distribution-specific package.

The latest *macroape* bundle can is always available at [http://autosome.ru/macroape/]. Advanced ruby users may check the last section of this manual for details on *macroape* gem package.

All tools in *macroape* bundle provide detailed help messages if executed without parameters (or if "-h" or "--help" is specified in the command line).

Introduction

Typical methods of comparing PWMs are based on direct comparison of elements, for instance by comparing matrices column by column (where different columns correspond to different positions of a binding site). On the other hand, in applications PWM is used as TFBS model to identify “binding sites” by scanning a given text and identifying words having PWM scores no less than a preselected threshold. So, in reality a TFBS model is related to the set of words scoring no less than the given threshold for the given PWM. It is desirable to construct a similarity measure for TFBS models according to the similarity between word sets recognized by the matrices with given thresholds, rather than according to similarity between matrices per se. Moreover, comparison-by-elements strategy requires the matrices to have algebraically comparable values (either frequencies or specifically scaled weights) which is not necessary if sets of recognized TFBS are compared.

Here we present a similarity measure which directly accounts for the similarity of recognized word sets. This measure does not require PWM elements to be algebraically comparable and so it can be used to compare weight matrices constructed by different normalization / conversion strategies.

Let us have a position weight matrix of length w. The whole set of ACGT-alphabet words of length w will be called *the dictionary* of size $N=4^w$. For a fixed threshold level t one can calculate the fraction of the dictionary (i.e. the number of words n) scoring no less than the threshold. We will call the value of n / N as the motif P-value.

Suppose we have 2 PWMs m_1, m_2 of length w and some P-value levels p_1, p_2. For m_1 and m_2 we can estimate the thresholds t_1 and t_2 corresponding to p_1, p_2. Having PWMs with the corresponding thresholds we can estimate the fraction f of the dictionary recognized by both models, i.e. the size of the set of words scoring no less than t_1 on m_1 and no less than t_2 on m_2.

Moreover one can construct the Jaccard index $J = \mathbf{A}\cap\mathbf{B} \,/\, \mathbf{A}\cup\mathbf{B}$ where **A** and **B** are sets of words recognized by m_1 and m_2 with the thresholds t_1 and t_2. If necessary one also can construct a Jaccard distance as $d(\mathbf{A},\mathbf{B}) = 1 - J$.

In the general case we have two PWMs of different widths, unknown optimal mutual alignment and orientation. For each possible alignment shift and orientation the matrices can be extended to the same length by adding zero-columns (not affecting either score or threshold) and then compared as the two models of the same width. Then one can determine the optimal shift and orientation by selecting the case with the highest Jaccard similarity. More formal and detailed explanation can be found in the corresponding *macroape* paper.

Basic tools

motifs "pat"-format description

All tools described below use the following matrix file format (each binding site position corresponds to separate line):

```
some_header
pos1_A_weight pos1_C_weight pos1_G_weight pos1_T_weight
..
posw_A_weight posw_C_weight posw_G_weight posw_T_weight
```

The number of the lines corresponds to the PWM width. Some real-life examples are provided within the *macroape* package.

All tools except `preprocess_collection` print results into stdout (standard output). `preprocess_collection` yields result to the output file. `preprocess_collection` and `scan_collection` print progress information to the stderr stream.

Output generally consists of two line types. Lines starting with '#' character (comment) show input parameter values and descriptions. The results are presented in non-commented lines.

find_threshold.rb

Stand-alone tool to search for the threshold corresponding to a given P-value for a given PWM. This scripts requires a PWM and a P-value as input and returns a threshold for which the set of words scoring with this PWM above the given threshold has the aggregated probability equal to the given P-value. The program can process a set of P-values, and return a set of thresholds. This tool implements the algorithm similar to that implemented in TFM-Pvalue software of Helen Touzet [http://bioinfo.lifl.fr/TFM/TFMpvalue/] with fixed discretization level (see "PWM discretization" section below).

Example (motif file KLF4_f2.pat, P-value of 0.001 and 0.0005):

```
ruby find_threshold.rb motifs/KLF4_f2.pat 0.001 0.0005
```

Output:

```
# V: discretization value
# PB: P-value boundary
# V = 10000
# PB = lower
#
# P: requested P-value
# AP: actual P-value
# W: number of recognized words
# T: threshold
# P      AP         W        T
0.0005  0.000499725341796875    524.0   5.24071
0.001   0.00099945068359375     1048.0  4.17189
```

For a more precise result one can use "-d" command line key like "-d 100000" to explicitly set the discretization level for PWM elements (see the "PWM discretization" section below for details). The discretization level of 10^5 corresponds to the precision of PWM elements up to 5 decimal places. A larger number of decimal places results in the increased precision and computational time. The default setting of 10000 gives reasonable "time-precision" tradeoff.
NOTE! By default `find_threshold` looks for threshold large enough to obtain P-value not greater than requested (lower boundary for P-value).

find_pvalue.rb

A stand-alone tool to find the P-value corresponding to a given threshold level for a given PWM.

Example (motif file `KLF4_f2.pat`, thresholds of 4.1719 and 5.2403):

```
ruby find_pvalue.rb  motifs/KLF4_f2.pat  4.1719  5.2403
```

Output:

```
# V: discretization value
# V = 10000
#
# T: threshold
# W: number of recognized words
# P: P-value
# T     W       P
4.1719  1048.0  0.00099945068359375
5.2403  524.0   0.000499725341796875
```

eval_similarity.rb

Stand-alone tool to compute the similarity for two given motifs with given thresholds or given P-value level. Uses P-value of 0.0005 by default.

Example (rather similar motifs KLF4_f2 and SP1_f1):

```
ruby eval_similarity.rb motifs/KLF4_f2.pat motifs/SP1_f1.pat
```

Output:

```
# V: discretization
# P: requested P-value
# PB: P-value boundary
# V = 10.0
# P = 0.0005
# PB = upper
#
# S: similarity
# D: distance (1-similarity)
# L: length of the alignment
# SH: shift of the 2nd PWM relative to the 1st
# OR: orientation of the 2nd PWM relative to the 1st
# A1: aligned 1st matrix
```

```
# A2: aligned 2nd matrix
# W: number of words recognized by both models (model = PWM +
threshold)
# W1: number of words and recognized by the first model
# P1: P-value for the 1st matrix
# T1: threshold for the 1st matrix
# W2: number of words recognized by the 2nd model
# P2: P-value for the 2nd matrix
# T2: threshold for the 2nd matrix
S       0.24382446963092125
D       0.7561755303690787
L       11
SH      -1
OR      direct
A1      .>>>>>>>>>>
A2      >>>>>>>>>>>
W       839.0
W1      2104.0
P1      0.0005016326904296875
T1      5.8
W2      2176.0
P2      0.000518798828125
T2      5.6
```

Example (the same motif SP1_f1 in opposite orientations):

```
ruby eval_similarity.rb motifs/SP1_f1_revcomp.pat motifs/SP1_f1.pat
```

Output:

```
# V: discretization
# P: requested P-value
# PB: P-value boundary
# V = 10.0
# P = 0.0005
# PB = upper
#
# S: similarity
# D: distance (1-similarity)
# L: length of the alignment
# SH: shift of the 2nd PWM relative to the 1st
# OR: orientation of the 2nd PWM relative to the 1st
# A1: aligned 1st matrix
# A2: aligned 2nd matrix
# W: number of words recognized by both models (model = PWM +
threshold)
# W1: number of words and recognized by the first model
# P1: P-value for the 1st matrix
# T1: threshold for the 1st matrix
# W2: number of words recognized by the 2nd model
# P2: P-value for the 2nd matrix
# T2: threshold for the 2nd matrix
```

```
S       1.0
D       0.0
L       11
SH      0
OR      revcomp
A1      >>>>>>>>>>>
A2      <<<<<<<<<<<
W       2176.0
W1      2176.0
P1      0.000518798828125
T1      5.6
W2      2176.0
P2      0.000518798828125
T2      5.6
```

Example (significantly different motifs SP1_f1 and GABPA_f1):

```
ruby eval_similarity.rb motifs/SP1_f1.pat motifs/GABPA_f1.pat
```

Output:

```
# V: discretization
# P: requested P-value
# PB: P-value boundary
# V = 10.0
# P = 0.0005
# PB = upper
#
# S: similarity
# D: distance (1-similarity)
# L: length of the alignment
# SH: shift of the 2nd PWM relative to the 1st
# OR: orientation of the 2nd PWM relative to the 1st
# A1: aligned 1st matrix
# A2: aligned 2nd matrix
# W: number of words recognized by both models (model = PWM +
threshold)
# W1: number of words and recognized by the first model
# P1: P-value for the 1st matrix
# T1: threshold for the 1st matrix
# W2: number of words recognized by the 2nd model
# P2: P-value for the 2nd matrix
# T2: threshold for the 2nd matrix
S       0.005720576093936336
D       0.9942794239060637
L       14
SH      1
OR      direct
A1      >>>>>>>>>>>...
A2      .>>>>>>>>>>>>>
W       1559.0
```

```
W1      139264.0
P1      0.000518798828125
T1      5.6
W2      134820.0
P2      0.0005022436380386353
T2      4.5
```

Note! By default `eval_similarity` selects the threshold corresponding to the P-value not less than requested (upper boundary). This is different from `find_threshold` which uses lower boundary for P-value.

It is very important to select upper P-value boundary for short PWMs. In case of given low P-values they can recognize no words at all (so the Jaccard measure may have zero numerator and zero denominator). For reasonable threshold levels both upper and lower boundaries produce very close similarity values.

Nevertheless, one can override this behavior with '--boundary lower' option. In such a case if any of supplied PWMs recognizes no words for a selected P-value, then similarity can not be correctly determined and *macroape* will report the similarity value of -1.

This also affects the search through a model collection (see below).

NOTE! For `scan_collection` tool this affects only a query PWM. The same option should be specified with `preprocess_collection` when preprocessing a PWM collection

eval_alignment.rb

Stand-alone tool to calculate the Jaccard index for two given motifs and a given P-value level using predefined motif alignment. Uses P-value of 0.0005 by default.

Example (rather similar motifs KLF4_f2 and SP1_f1 at optimal alignment):

```
ruby eval_alignment.rb motifs/KLF4_f2.pat motifs/SP1_f1.pat -1 direct
```

Output:

```
# V: discretization
# P: requested P-value
# PB: P-value boundary
# V = 10.0
# P = 0.0005
# PB = upper
#
# S: similarity
# D: distance (1-similarity)
# L: length of the alignment
# SH: shift of the 2nd PWM relative to the 1st
# OR: orientation of the 2nd PWM relative to the 1st
# A1: aligned 1st matrix
# A2: aligned 2nd matrix
# W: number of words recognized by both models (model = PWM + threshold)
# W1: number of words and recognized by the first model
# P1: P-value for the 1st matrix
# T1: threshold for the 1st matrix
```

```
# W2: number of words recognized by the 2nd model
# P2: P-value for the 2nd matrix
# T2: threshold for the 2nd matrix
S       0.24382446963092125
D       0.7561755303690787
L       11
SH      -1
OR      direct
A1      .>>>>>>>>>>
A2      >>>>>>>>>>>
W       839.0
W1      2104.0
P1      0.0005016326904296875
T1      5.8
W2      2176.0
P2      0.000518798828125
T2      5.6
```

Example (rather similar motifs KLF4_f2 and SP1_f1 at completely wrong alignment):

```
ruby eval_alignment.rb motifs/KLF4_f2.pat motifs/SP1_f1.pat 3 revcomp
```

Output:

```
# V: discretization
# P: requested P-value
# PB: P-value boundary
# V = 10.0
# P = 0.0005
# PB = upper
#
# S: similarity
# D: distance (1-similarity)
# L: length of the alignment
# SH: shift of the 2nd PWM relative to the 1st
# OR: orientation of the 2nd PWM relative to the 1st
# A1: aligned 1st matrix
# A2: aligned 2nd matrix
# W: number of words recognized by both models (model = PWM + threshold)
# W1: number of words and recognized by the first model
# P1: P-value for the 1st matrix
# T1: threshold for the 1st matrix
# W2: number of words recognized by the 2nd model
# P2: P-value for the 2nd matrix
# T2: threshold for the 2nd matrix
S       0.0
D       1.0
L       14
SH      3
OR      revcomp
A1      >>>>>>>>>>....
A2      ...<<<<<<<<<<<
W       0.0
W1      134656.0
P1      0.0005016326904296875
T1      5.8
W2      139264.0
P2      0.000518798828125
T2      5.6
```

Searching for similar motifs in a given motif collection

The comparison strategy does not require PWMs to be directly comparable (so PWMs can be constructed from position count matrices, PCMs, using completely different approaches). Still it is prudent to use similarly processed matrices for collection and a query motif.

For example, matrix of positional counts (PCM) can be transformed into PWM according to the formula used in **[Lifanov2003]**:

$$S_{\alpha,j} = ln\frac{x_{\alpha,j}+aq_{\alpha}}{(W+a)q_{\alpha}}$$

where *W* is the total weight of the alignment (or the number of aligned words), *a* is the pseudocount value selected as the *ln(W)*, *S* is the PWM element, *x* is the corresponding PCM (position count matrix) element at *j*-th column, and *q* is the background probability of nucleotide letter *α*.

macroape can preprocess collections to be then used to look for motifs similar to a given query. We provide two instances of the HOCOMOCO-AD collection of TFBS models for human TFs. The PWMs are normalized to the uniform and hg19 background distributions (using the formula above).

preprocess_collection.rb

This tool is intended to process the motif collection (presented as a folder containing separate file for each motif) and create a yaml-file containing discretized matrices and corresponding thresholds for a given P-value level.

Example:

```
ruby preprocess_collection.rb ./motifs collection.yaml
```

This will produce the `collection.yaml` file containing discretized PWMs along with the thresholds for the PWM collection in folder `./motifs` using default P-value of 0.0005. Two discretization levels are used by default: 1 for rough processing and 10 for precise processing.

In order to change discretization levels you can use "-d" option with two parameters, rough_discretization and precise_discretization:

```
ruby preprocess_collection.rb ./motifs collection.yaml -d 1,100
```

If you plan to search for similar models on different P-value levels you should use the "-p" option with the list of required P-value levels like:

```
ruby preprocess_collection.rb ./motifs collection.yaml -p
0.001,0.0005,0.0001
```

It takes about ten minutes to preprocess the whole collection of ~400 PWMs with default parameters using 1.5 GHz CPU.

We provide precalculated yaml-files for the HOCOMOCO-AD collection of PWMs constructed using uniform and hg19 background nucleotide distributions; yaml-files were generated by the following commands:

```
ruby preprocess_collection.rb HOCOMOCO_AD_uniform
hocomoco_ad_uniform.yaml -p 0.001,0.0005,0.0001,0.00005,0.00001

ruby preprocess_collection.rb HOCOMOCO_AD_hg19 hocomoco_ad_hg19.yaml -p
0.001,0.0005,0.0001,0.00005,0.00001
```

scan_collection.rb

This tool uses a preprocessed collection to find PWMs similar to a given query. The tool returns a list of all PWMs in the collection with corresponding similarity levels comparing to the query PWM. List is sorted by similarity in descending order so the PWMs similar to the query are located at the top of the list.

NOTE! The shift and orientation are reported for PWMs from the collection relative to the query PWM.

The tool will print processed matrices to the STDERR and the resulting list to the STDOUT (so one can append "> output.txt" to the end of the command line to save the result into a plain text file).

Example (search for motifs similar to KLF4_f2, HOCOMOCO collection with uniform background), should take ~1-2 minutes:

```
ruby scan_collection.rb motifs/KLF4_f2.pat hocomoco_ad_uniform.yaml
```

Output (STDOUT):

```
# MS: minimal similarity to output
# P: P-value
# PB: P-value boundary
# V: discretization value
# MS = 0.05
# P = 0.0005
# PB = upper
# V = 1
#
# motif similarity      shift   overlap orientation
KLF4_f2 1.0     0       10      direct
SP1_f2  0.26782003264743703     -6      10      direct
KLF1_f1 0.26633986928104575     -1      10      direct
SP1_f1  0.2580456407255705      -1      10      direct
SP3_f1  0.22040908979266507     -5      10      direct
ZIC1_f1 0.13428571428571429     0       9       direct
ZN148_si        0.12878506231454379     -6      9       direct
WT1_f1  0.12858931552587646     -3      10      direct
KLF3_f1 0.10248264322732754     -4      10      direct
```

```
EGR4_f1 0.0688230008984726      -1      10      direct
SP2_si  0.06371381046574356     -4      10      direct
EGR3_f1 0.06049086691229506     -4      7       direct
EGR2_si 0.05626489836605556     -4      7       direct
ZN219_f1        0.051975747438845914    -2      10      direct
```

One can also use the two-pass search mode when the top of the list is additionally reprocessed using a more precise discretization level. Second pass is executed only if "--precise [min_similarity=0.01]" key is specified. The precise search will recheck only the PWMs similar to the query with a similarity no less than [min_similarity]. The results of the second pass will be marked by asterisk(*) in the output.

One can also specify similarity cutoff with option -c <similarity cutoff> to discard comparison results with the resulting similarity less than a given value. In order to print comparison results for all PWMs in collection, one should specify --all option.

Example (search PWMs similar to KLF4_f2, uniform normalization, extended precision for the most similar PWMs), should take ~3 minutes for 1.5 GHz CPU:

```
ruby scan_collection.rb motifs/KLF4_f2.pat hocomoco_ad_uniform.yaml --precise
```

Output (STDOUT):

```
# MS: minimal similarity to output
# P: P-value
# PB: P-value boundary
# VR: discretization value, rough
# VP: discretization value, precise
# MP: minimal similarity for the 2nd pass in 'precise' mode
# MS = 0.05
# P = 0.0005
# PB = upper
# VR = 1
# VP = 10
# MP = 0.05
#
# motif similarity      shift   overlap orientation     precise mode
KLF4_f2 1.0     0       10      direct  *
KLF1_f1 0.26124515936848375     -1      10      direct  *
SP1_f1  0.24382446963092125     -1      10      direct  *
SP1_f2  0.2066482768325595      -6      10      direct  *
SP3_f1  0.19157407286598827     -5      10      direct  *
WT1_f1  0.126410686671273       -3      10      direct  *
KLF3_f1 0.11028473004279815     -4      10      direct  *
ZN148_si        0.09694303022157706     -6      9       direct  *
SP2_si  0.06967957253814294     -4      10      direct  *
ZIC1_f1 0.06158357771260997     0       9       direct  *
EGR4_f1 0.059941520467836254    -1      10      direct  *
```

```
EGR2_si 0.055004913225474496    -4      7       direct  *
EGR3_f1 0.051192488841477736    -4      7       direct  *
```

To find similar PWMs using a particular P-value level one should use the "-p" option. In order to process this request the yaml-file of the collection should contain precalculated thresholds for the selected P-value level (you can always reprocess the collection to include the selected P-value level using preprocess_collection with the "-p" key).

Example (should take ~4 minutes for 1.5GHz CPU):

```
ruby scan_collection.rb motifs/KLF4_f2.pat hocomoco_ad_uniform.yaml -p
0.001 -c 0.06 --precise 0.1
```

Output (STDOUT):

```
# MS: minimal similarity to output
# P: P-value
# PB: P-value boundary
# VR: discretization value, rough
# VP: discretization value, precise
# MP: minimal similarity for the 2nd pass in 'precise' mode
# MS = 0.06
# P = 0.001
# PB = upper
# VR = 1
# VP = 10
# MP = 0.1
#
# motif similarity      shift   overlap orientation     precise mode
KLF4_f2 1.0     0       10      direct  *
SP1_f1  0.28505023241865346     -1      10      direct  *
KLF1_f1 0.2776963657678781      -1      10      direct  *
SP1_f2  0.2433013733784068      -6      10      direct  *
SP3_f1  0.22703529219653049     -5      10      direct  *
WT1_f1  0.14997613445096677     -3      10      direct  *
KLF3_f1 0.13269732789427957     -4      10      direct  *
ZN148_si        0.11645522380512197     -6      9       direct  *
EGR4_f1 0.09662000682826903     -1      10      direct  .
ZIC1_f1 0.08305166586190246     0       9       direct  *
SP2_si  0.07963212355722984     -4      10      direct  .
EGR3_f1 0.07585601954825977     -4      7       direct  .
EGR2_si 0.06989664406016166     -4      7       direct  .
ZIC2_f1 0.06682941714983216     0       9       direct  .
ZN219_f1        0.06496764822395353     -2      10      direct  .
KLF8_f1 0.06072493331241174     -1      8       direct  .
```

Advanced command-line options

Additional options for *macroape* tools:

--help: show information on command-line parameters

-b <probA,probC,probG,probT>: set the background nucleotide composition
--max-hash-size <size>: set the internal hash size limit
--pcm: provide motif matrixes as PCM instead of PWM, conversion to be done internally

By default the maximum hash size is limited by 10^7. This can be not enough to process an extremely high discretization level (see below) or specifically arranged artificial matrices.

Additional "--max-2d-hash-size <size>" can be used for **eval_similarity**, **eval_alignment** and **scan_collection** tools. It sets the internal two-dimensional hash size used for PWM comparison. By default the total size is limited by 10^4.

The error message "Hash overflow in PWM/PWMCompare" means the maximum Hash size should be increased by "--max-hash-size/--max-2d-hash-size" key.

The "-b" key can be used to supply a given background nucleotide composition so the words in the dictionary became weighted according to corresponding probabilities to be generated by *i.i.d.* random model.

NOTE! The background nucleotide composition for searching similar motifs in a collection is stored within the collection yaml-file (so you should set the background during collection preprocessing via **preprocess_collection**).

NOTE! The reverse complementary transform can be necessary to optimally align matrices, thus the background nucleotide composition for matrix comparison (**eval_similarity** and **preprocess_collection** tools) should be symmetrical, i.e. p(A) = p(T) and p(C) = p(G).

The order of nucleotides is assumed alphabetical, so the "-b 0.2,0.3,0.3,0.2" will correspond to p(A,C,G,T) = {0.2, 0.3, 0.3, 0.2}. In case of a given background model tools print out not the number of words but the probability to randomly draw a word scoring no less than the threshold from the complete dictionary of words weighted according to the given background model.

All tools except find_pvalue have additional option:

--boundary <lower|upper>: prefer threshold yielding actual P-value below/above than the requested P-value

Since PWM P-values have discrete distribution a given P-value can be achieved only approximately. For model comparison by default we use the lower boundary for the threshold (so even at low given P-values PWMs recognize some words and thus the models can be compares). If searching for a threshold corresponding to the given P-value we report the upper boundary of the threshold by default.

`preprocess_collection` and `scan_collection` have additional option "--silent" which disables the output of progress information (printed to stderr by default).

Experimental features

Usually tools load PWMs from given fileы. All tools also can load input matrices from the standard input (stdin). In order to load a matrix from the standard input you should use a placeholder ".stdin" instead of a filename, like:

```
cat motifs/KLF4_f2.pat | find_threshold .stdin 0.0005 0.001
cat motif1.pwm motif2.pwm | eval_similarity .stdin .stdin
cat motif2.pwm | eval_similarity motif1.pwm .stdin
```

PWM discretization

Following the general idea described in **[Touzet2007]** we can effectively calculate the P-value for a given PWM having fixed precision and a given threshold value. The algorithm of Touzet efficiently processes matrices with integer elements, so the real value matrices are transformed into integer value matrices by multiplying on discretization constant and truncating the decimals. Effectively this is similar to rounding up real value PWMs leaving only the fixed number of decimal places. The higher discretization level will result in a more accurate P-value calculation and an increased computational time.

Please note, that in contrast to the original Touzet algorithm here we applying "ceil" operation to the matrix elements (instead of "floor" in the original paper of Touzet). This allows us to have a strict upper boundary of the threshold for a given P-value.

We use the default discretization level of 10000 to perform calculation accuracy up to four significant digits for single-PWM tools. The straightforward discretization by rounding up to the nearest integer is used by default for a fast and rough search through the motif collection. The default level of 10 (one decimal place) is used for a more precise search of similar motifs.

Thus in our case discretization is the transformation as follows: discretized *S* is *S* multiplied by discretization level V and rounded up to the nearest integer value.

Example:

S = 1.6734
discretization V=1 → discretized *S* = ceil(1.6734) = 2
discretization V=10 → discretized *S* = ceil(16.734) = 17
discretization V=100 → discretized *S* = ceil(167.34) = 168

Discretization will generally preserve the word score ranking with the common exception for words that would obtain identical scores. The main advantage of the discretization is decreasing of the number of possible scores so the set of all possible scores can be enumerated more effectively.

macroape ruby gem

macroape is distributed as a standalone package. For advanced ruby users a gem installation is also possible.

In order to install gem one should use the following command:

```
gem install macroape
```

This version is more convenient, but behave slightly different:

Tools from the rubygem can be executed from any folder:

```
tool_name <arguments>
```

(omitting ruby and .rb, "ruby tool_name.rb <arguments>" won't work).

macroape gem version provides several additional tools like `align_motifs` to align several motifs relative to the first one. Also gem version installs 'bioinform' gem as a dependency (less documented but can be also useful too). For more information take a look at

https://github.com/prijutme4ty/macroape

and

https://github.com/prijutme4ty/bioinform

References

[Touzet2007] *Algorithms Mol Biol*. 2007 Dec 11;2:15. Efficient and accurate P-value computation for Position Weight Matrices. Touzet H, Varré JS.

[Pape2008] *Bioinformatics.* 2008 Feb 1;24(3):350-7. Epub 2008 Jan 2. Natural similarity measures between position frequency matrices with an application to clustering. Pape UJ, Rahmann S, Vingron M.

[Lifanov2003] *Genome Res*. 2003 Apr;13(4):579-88. Homotypic regulatory clusters in *Drosophila*. Lifanov AP, Makeev VJ, Nazina. AG, Papatsenko DA.
